# Fulminant Liver Failure Associated with Abdominal Crush Injury in an Eleven-Year Old: A Case Report

**DOI:** 10.1155/2013/524371

**Published:** 2013-10-22

**Authors:** Erin Gordon, Sameer Kamath

**Affiliations:** Department of Pediatrics, Division of Critical Care, University of Iowa Children's Hospital, Iowa City, IA 52242, USA

## Abstract

An 11-year-old obese male was involved in an all-terrain vehicle rollover accident. He had elevated transaminase levels along with a lactic acidosis. The imaging studies did not reveal any major intra-abdominal or thoracic injuries. The physical exam was unremarkable. The patient had an unremarkable PICU course and was transferred to the floor the next day. Within 24 hours of his transfer, he was noted to have interval worsening in liver function tests. He developed fulminant liver failure (FLF), renal failure, and encephalopathy. An ultrasound of the liver revealed increased echogenicity in the right lobe with focal sparing. Patient was listed for transplant. Investigations into any underlying medical cause of FLF were negative. Liver failure was presumed to be related to ischemia/reperfusion injury of the liver. The renal failure was due to rhabdomyolysis and was supported with renal replacement therapy. Patient received supportive care for FLF and was noted to have significant recovery of liver and renal function with time. He was discharged home after a 3-week hospitalization. Patients with crush abdominal injuries and elevated transaminase levels without evidence of parenchymal liver disruption may need to be closely monitored for liver failure related to ischemia reperfusion.

## 1. Introduction

Acute liver failure is characterized by the rapid development of severe liver injury with impaired synthetic function and hepatic encephalopathy in a patient without obvious, previous liver disease. Since the liver is capable of regeneration to a large extent, fulminant liver failure in principle may resolve with complete recovery. The decision for transplantation depends upon the likelihood of a spontaneous recovery. The indications for liver transplantation after trauma include uncontrolled hemorrhage, severe grade 4-5 injury resulting in liver parenchyma disruption, irreversible liver failure, and life-threatening postreperfusion injury. The requirement for liver transplantation after major liver trauma is rare with 19 cases reported in the literature with variable outcome [1]. FLF following abdominal trauma without hepatic parenchymal disruption has yet to be described in the pediatric population. In this case report, we describe an 11-year-old obese male with fulminant hepatic failure following a crush injury of the abdomen thought to be related to hepatic ischemia/reperfusion (I/R) injury.

## 2. Case Description

**Figure 1:**
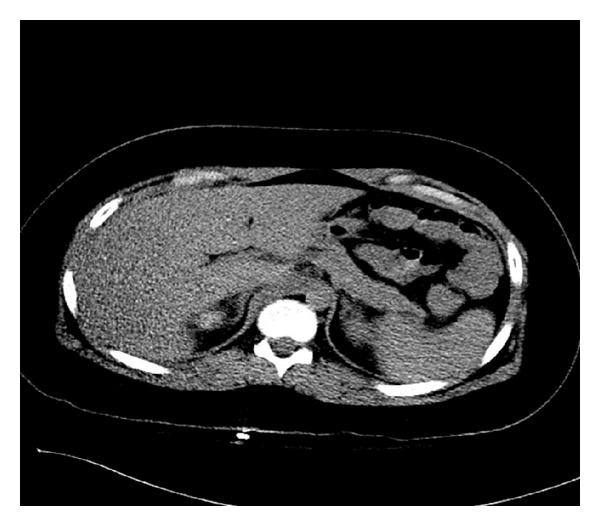
Abdominal CT scan showing no hepatic parenchymal injury.

An 11-year-old obese boy (weight 84 kilograms, height 1.52 meters with BMI of 36.17 kg/m^2^) was involved in an all-terrain vehicle (ATV) rollover accident. He was extricated by his grandfather and was taken by emergency medical services to the local emergency room. He received 2 liters of lactated ringers (LR) and underwent radiographic studies and laboratory tests. The laboratory evaluation at the local emergency room was remarkable for mild hypokalemia, elevated transaminase levels, and mild elevation in pancreatic enzymes. Head, chest, and Abdominal CT scans were unremarkable for major organ injury or bleeding. The patient was then transferred to our emergency room for further evaluation. The physical exam was remarkable for obesity, mild bruising over the abdomen, and an imprint from the ATV handle bar in the subcostal region. Outside laboratory results were reviewed and repeat laboratory tests at our center revealed a drop in hemoglobin from 15 to 10 g/dL with lactic acidosis. A repeat CT of the abdomen and chest was obtained to identify concealed blood loss, but again negative (Figure 1). The patient was transferred to the Pediatric Intensive Care Unit (PICU) for serial monitoring of abdominal exams, hemoglobin/hematocrit, and hemodynamic status.

On admission to the PICU, he was noted to be hemodynamically stable. He had mild discomfort in the right upper quadrant of the abdomen with the imprint of the ATV handle bar and mild bruising. The cardiopulmonary exam was unremarkable. The patient was neurologically normal and denied any pain/tenderness along the spine. The patient's hemoglobin/hematocrit, hemodynamics, and liver enzymes remained stable overnight. His lactic acidosis had completely resolved with a peak venous lactate of 7 mEq/L. He was transferred to the floor on pediatric surgery service on hospital day (HD) 2 for further care until discharge from the hospital. He was transitioned to an enteral diet and intravenous fluids were discontinued. On HD 3, he was noted to have lethargy secondary to significant hypoglycemia (blood glucose level of 14 gm/dL). His mental status did not normalize, despite the correction of the hypoglycemia (blood glucose level of 78 gm/dL). His liver enzymes had worsened in the interim with worsening renal function and a decrease in urine output over the past 12–18 hours (Table 1). He was transferred back to the PICU urgently for monitoring of blood glucose and mental status.

**Figure 2:**
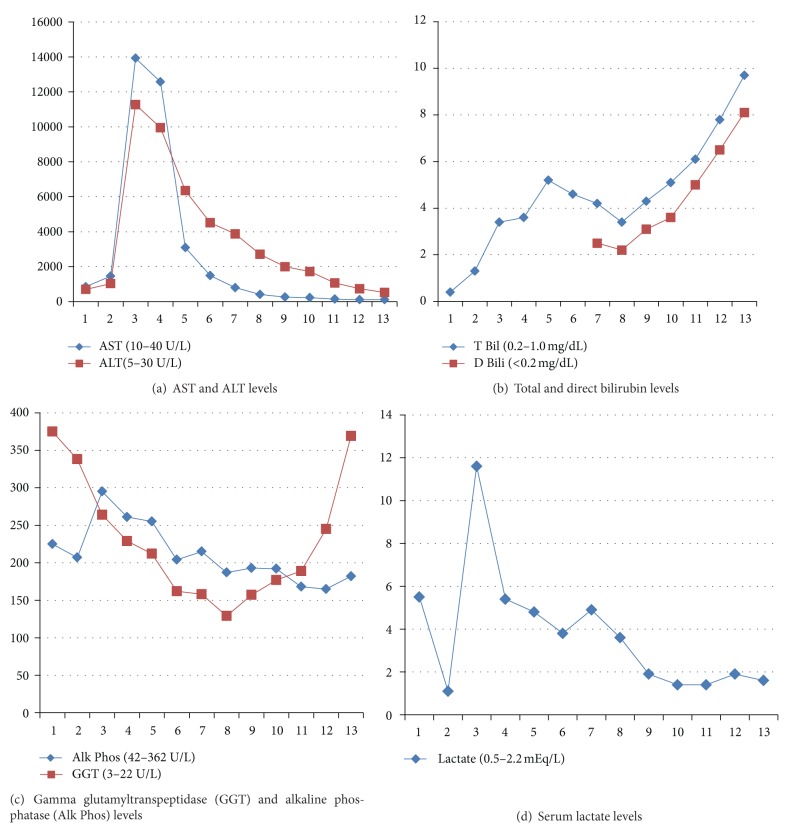
Laboratory trends from the emergency room to hospital day 13: Aspartate transaminase (AST) and alanine transaminase (ALT) (a); total and direct bilirubin (b), gamma glutamyltranspeptidase (GGT) and alkaline phosphatase (Alk Phos) (c), and serum lactate (d).

On readmission, the patient was noted to have altered mental status with confusion and delirium consistent with stage 2 hepatic encephalopathy. The abdominal exam was unremarkable for any tenderness or distension and bowel sounds were audible. He was hemodynamically stable. Patient was noted to have rhabdomyolysis with an elevated creatine kinase level of 36,399 U/L (*N* = 40–200 U/L) with interval development of renal failure (blood urea nitrogen (BUN) 26 mg/dL and creatinine (Cr) 1.5 mg/dL). Liver enzymes were significantly worse on readmission with a 10-fold increase in aspartate transaminase (AST) and alanine transaminase (ALT) with interval reappearance of significant lactic acidosis (Table 1). The patient was noted to be severely coagulopathic with a prothrombin time (PT) of 90 seconds, international normalized ratio of 9.9, and partial thromboplastin time (PTT) of 40 seconds. He received cryoprecipitate and fresh frozen plasma for severe coagulopathy due to FLF prior to the placement of a peripherally inserted central line for stable venous access. Hypoglycemia improved and stabilized with a glucose infusion rate of 6–8 mg/kg/min with gradual improvement in lactic acidosis (Figure 2(d)). Serum ammonia was noted to be 112 mmol/L. The pediatric gastroenterology, hepatology, and transplant consulting services were notified. A thorough evaluation of the potential causes of FLF was initiated based on recommendations made by the subspecialists. He had received acetaminophen during his hospital stay. The total dose administered was well below toxic range and the level obtained was therapeutic. In spite of this finding, intravenous N-acetylcysteine therapy was initiated after discussion with all specialists involved to replace hepatic glutathione stores in hopes of scavenging reactive oxygen species. 

There was significant deterioration in his neurologic status with progression to stage III hepatic encephalopathy overnight (HD 4) along with respiratory distress due to fluid overload and renal failure. Urgently, he was intubated followed by placement of invasive lines for hemodynamic monitoring and initiation of renal replacement therapy. He received one dose of activated factor VII to limit any hemorrhagic complications from the invasive procedures (arterial line placement, central line insertion, and temporary hemodialysis catheter placement) given continued moderate-to-severe coagulopathy. He received aggressive supportive cares for his acute liver failure. The patient was listed for a liver transplant as a status 1 A. 

The patient continued to receive supportive care over the course of the next week with stabilization of respiratory status, improvement in renal function, and improvement in his coagulopathy and liver failure (Figure 2). He was extubated on HD 10 and tolerated the same well. He was removed from the transplant list given continued improvement in liver synthetic function and was transferred to the floor on HD 12. His renal support was discontinued on HD 15 and the dialysis catheter was removed on HD 18. He was discharged home on HD 21 with scheduled followup arranged with gastroenterology, nephrology, and pediatric surgery. Peak abnormal laboratory values and values at the time of discharge for AST, ALT, bilirubin, BUN, and creatinine are listed in Table 2. Investigations to assess any underlying metabolic, autoimmune, infectious, or genetic causes of liver failure were unremarkable. The patient's FLF was attributed to the abdominal crush injury that may have resulted in I/R of the liver.

At followup 2 weeks and 1 month from discharge, the liver and renal function tests were normal and patient was noted to be doing well without any sequelae from his injury and subsequent fulminant liver failure.

## 3. Discussion

### 3.1. Fulminant Liver Failure

 Despite some degree of protection from the overlying rib cage, the liver is susceptible to injury from blunt abdominal trauma [2]. Liver trauma represents 1.2–4.6% of all trauma-related hospitalizations. It is more common in males and during the second and third decades of life. It is associated with 10–30% mortality. Nonoperative management of blunt liver injury is associated with lower hospital stay, transfusion needs, and rate of intra-abdominal infections [3].

Our patient had elevation in AST and ALT suggesting liver injury from the accident but no parenchymal lesion or vascular disruption was noted on CT scan (Figure 1). Clinically, he appeared well and denied significant abdominal pain on repeated evaluation in the emergency room and the PICU. He was transferred to the floor after a 24-hour period of observation in the PICU with stable vitals, reassuring exam, resolved lactic acidosis, and stable LFTs.

Our patient developed FLF and required readmission to the PICU about 48 hours after his injury. The mechanism of injury was noted to be crush from the ATV rollover. The ATV handlebar imprint was notably in the subcostal region. It is possible that the weight of the ATV resulted in hepatic ischemia caused by compression of the portal vein and hepatic artery, which resolved as soon as he was extricated. Further review of the abdominal CT scans did not reveal any vascular injury or thrombosis. Bedside ultrasound was done when the patent was readmitted and revealed increased echogenicity consistent with fatty infiltration in the right lobe with focal sparing with good flow in the portal vein and hepatic artery. However, on repeat evaluation, the patient has no radiographic evidence of fatty liver disease. The pediatric gastroenterologists have not diagnosed him with fatty liver disease at this time. The liver synthetic function recovered spontaneously and the patient needed supportive care for his FLF for about 2 weeks. The thorough evaluation into the cause of FLF (infection, autoimmune, and genetic/metabolic) was negative thus making I/R injury to be the most likely explanation of his clinical course.

I/R injury occurs when the liver is transiently deprived of oxygen and then is reoxygenated. When hepatic blood supply is temporarily interrupted, which is thought to be the case in this report of abdominal trauma, it results in warm ischemia. Warm I/R injury involves the activation of the immune system and is dominated by hepatocellular injury. The initial phase of injury (early phase) is marked by activation of immune cells and production of oxidant stress; this phase typically occurs within 2 hours following reperfusion. The late phase of injury is mediated by neutrophil accumulation and hepatocellular injury and typically occurs 6–48 hours after reperfusion. It has been associated with low flow states, surgical procedures, and during organ procurement for transplantation (cold ischemia) [4]. The pathogenesis of hepatic I/R injury is complex and includes activation of Kupffer cells, endothelial cells, leucocytes, and reactive oxygen species [5]. I/R injury is associated with histopathologic changes of cellular swelling, vacuolization, and leucocyte infiltration typically seen within 48 hours of reperfusion with normalization of liver architecture within 2 weeks of reperfusion [6]. Although several therapeutic interventions are suggested, none have been proven to be successful in humans. 

### 3.2. Lactic Acidosis

 Lactic acidosis following trauma may suggest inadequate tissue oxygen delivery and is often seen in association with severe hemorrhage. Clearance of lactate is decreased in patients with hepatic dysfunction [7]. LR contains sodium lactate. Our patient received 2 liters of LR at the outside emergency room and another 1 L in our institution. Lactic acidosis was noted despite any hemorrhage or evidence of inadequate tissue oxygen delivery which cleared after overnight observation in the PICU after his fluids were switched to 5% dextrose/half normal saline with potassium. In hindsight, his lactic acidosis was likely due to the exogenous lactate load in the setting of impaired lactate clearance secondary to hepatic dysfunction from ischemic injury sustained in the accident. The reappearance of lactic acidosis at readmission was likely representative of the degree of liver dysfunction from the I/R injury with inability to clear endogenous lactate. Significant lactic acidosis in the absence of inadequate tissue oxygen delivery following administration of LR may suggest liver dysfunction and should be appropriately monitored.

### 3.3. Renal Failure

We believe that the renal failure in our patient was due to a combination of rhabdomyolysis and contrast exposure. Crush injury is associated with rhabdomyolysis and renal failure due to acute tubular necrosis (ATN). Our patient had an abdominal crush injury. His clinical exam was very benign without any clinical signs of significant abdominal muscle injury. Patient's clinical wellness in the first 48 hours of hospitalization may have falsely reassured the team. It was only after he was re-admitted to the PICU with oliguria that a creatinine kinase level was obtained and found to be markedly elevated.

Intravenous contrast agents are a well-known cause of renal failure especially when other contributing factors coexist. Abdominal CT scan is the standard of care when intra-abdominal injury is suspected based on mechanism of injury and screening laboratory tests [2, 3]. Although our patient received intravenous fluids before intravenous contrast agents were administered, he may have been relatively intravascularly depleted contributing to contrast-induced renal injury.

The patient tolerated continuous renal replacement therapy very well and was transitioned to intermittent hemodialysis. He was noted to have adequate recovery of renal function in 2 weeks and at followup 1 month from his hospitalization he was noted to have normal renal function. Thus, obtaining a creatinine kinase level earlier in his course may have allowed therapies aimed at preventing acute tubular necrosis.

## 4. Conclusion

Abnormal AST and ALT in the absence of hepatic parenchymal disruption in abdominal crush injury may be related to transient hepatic ischemia. These patients may be at risk of FLF from reperfusion injuries. Lactic acidosis in the absence of inadequate tissue oxygen delivery following LR administration may suggest significant hepatic dysfunction. LR should be avoided in the setting of significant liver dysfunction to prevent development of lactic acidosis. Creatinine kinase should be obtained in trauma patients with a crush mechanism of injury to enable possible prevention of ATN. FLF due to ischemia/reperfusion injury often resolves spontaneously with supportive care, and liver transplantation is rarely needed.

## Figures and Tables

**Table 1:** Laboratory results at time of readmission to PICU.

Laboratory test	Result	Normal range
Alanine transaminase	11275 U/L	5–30 U/L
Aspartate transaminase	13926 U/L	10–40 U/L
Blood urea nitrogen	26 mg/dL	10–20 mg/dL
Creatinine	1.5 mg/dL	0.3–0.9 mg/dL
Ammonia	112 mmol/L	7–42 mmol/L
Prothrombin time	90 seconds	9–12 seconds
INR	9.9	1.1–1.3
Partial thromboplastin time	40 seconds	23–31 seconds
Creatinine kinase	36399 U/L	40–200 U/L

**Table 2:** Laboratory results: peak values and values at time of discharge.

Laboratory test	Peak value	Hospital day	Discharge value	Normal range
Total bilirubin	9.7 mg/dL	13	3.9 mg/dL	0.2–1 mg/dL
Direct bilirubin	8.1 mg/dL	13	2.7 mg/dL	<0.2 mg/dL
Aspartate transaminase	13926 U/L	3	98 U/L	10–40 U/L
Alanine transaminase	11275 U/L	3	129 U/L	5–30 U/L
Blood urea nitrogen	77 mg/dL	15	36 mg/dL	10–20 mg/dL
Creatinine	5.8 mg/dL	15	0.8 mg/dL	0.3–0.9 mg/dL

## Abbreviations

ATV: All-terrain vehicle
FLF: Fulminant liver failure
CT: Computed tomogram
PICU: Pediatric intensive care unit
I/R: Ischemia/reperfusion
HD: Hospital day
LFT: Liver function test
LR: Lactated ringers
AST: Aspartate transaminase
ALT: Alanine transaminase
BUN: Blood urea nitrogen
Cr: Creatinine.
